# Insights Into Spinal Dorsal Horn Circuit Function and Dysfunction Using Optical Approaches

**DOI:** 10.3389/fncir.2020.00031

**Published:** 2020-06-12

**Authors:** Erika K. Harding, Samuel Wanchi Fung, Robert P. Bonin

**Affiliations:** ^1^Department of Pharmaceutical Sciences, Leslie Dan Faculty of Pharmacy, University of Toronto, Toronto, ON, Canada; ^2^Department of Comparative Biology and Experimental Medicine, University of Calgary, Calgary, AB, Canada; ^3^University of Toronto Centre for the Study of Pain, University of Toronto, Toronto, ON, Canada

**Keywords:** spinal cord, dorsal horn, optogenetics, calcium imaging, *in vivo*, pain, somatosensation

## Abstract

Somatosensation encompasses a variety of essential modalities including touch, pressure, proprioception, temperature, pain, and itch. These peripheral sensations are crucial for all types of behaviors, ranging from social interaction to danger avoidance. Somatosensory information is transmitted from primary afferent fibers in the periphery into the central nervous system *via* the dorsal horn of the spinal cord. The dorsal horn functions as an intermediary processing center for this information, comprising a complex network of excitatory and inhibitory interneurons as well as projection neurons that transmit the processed somatosensory information from the spinal cord to the brain. It is now known that there can be dysfunction within this spinal cord circuitry in pathological pain conditions and that these perturbations contribute to the development and maintenance of pathological pain. However, the complex and heterogeneous network of the spinal dorsal horn has hampered efforts to further elucidate its role in somatosensory processing. Emerging optical techniques promise to illuminate the underlying organization and function of the dorsal horn and provide insights into the role of spinal cord sensory processing in shaping the behavioral response to somatosensory input that we ultimately observe. This review article will focus on recent advances in optogenetics and fluorescence imaging techniques in the spinal cord, encompassing findings from both *in vivo* and *in vitro* preparations. We will also discuss the current limitations and difficulties of employing these techniques to interrogate the spinal cord and current practices and approaches to overcome these challenges.

## Introduction

Our physical connection to the world through sensation is essential for our health and wellbeing. Somatosensation is a broad term encompassing many modalities including touch, pressure, proprioception, temperature, pain, and itch. It is through these peripheral sensations that we can recognize and remove ourselves from danger, to sense warmth or cold for thermoregulation, and to detect and respond to socially relevant physical gestures such as a gentle caress. An inability to detect physical sensations can be severely debilitating and lead to increased risk of injury and a shortened lifespan, as can be seen in patients with congenital insensitivity to pain (Nagasako et al., 2003).

Somatosensory information is first transduced in the peripheral nervous system by specialized receptors on primary afferent neurons. This information then travels along primary afferent fibers, whose cell bodies reside in the dorsal root ganglia, and into the dorsal horn of the spinal cord. Historically, primary afferent fibers have been classified by conduction velocity and degree of myelination into four types (Aα, Aβ, Aδ, and C), and it was originally believed that each type transmits different modalities of sensory information to the spinal dorsal horn (Roberts and Elardo, 1986; McGlone and Reilly, 2010).

C fibers terminate predominantly in laminae I–II, the outermost laminae, and transmit nociceptive information including noxious heat and noxious mechanical perturbations to tissue (Basbaum et al., 2009), as well as information related to itch and low threshold, pleasant mechanical stimuli (Olausson et al., 2002; Ikoma et al., 2006; Wooten et al., 2014; Huang et al., 2018). Aδ fibers transmit a mixture of noxious and innocuous tactile and cold information and terminate predominantly within laminae I and V, with a subset of Aδ fibers corresponding to low-threshold mechanosensation terminating within lamina III (Li et al., 2011; Arcourt et al., 2017; Koch et al., 2018). However, Aβ fibers carry the bulk of innocuous tactile information, including touch, vibration, texture, and pressure (Mackenzie et al., 1975; Basbaum et al., 2009). Aβ fibers transmit information *via* the dorsal column into the cuneate and gracile nuclei, and send a branch into the dorsal horn, terminating in laminae III–V (Basbaum et al., 2009; Niu et al., 2013). Proprioceptive information is transmitted predominantly by Aα fibers, which terminate widely throughout lamina IV–VI and the ventral horn, where they contribute to sensory-motor loops (Maxwell and Bannatyne, 1983; Mears and Frank, 1997; Maxwell and Riddell, 1999). It should be noted that there is increasing evidence that this classification of primary afferent types by conduction velocity and modality of information is not as discrete as once believed, and there are many exceptions to these general rules. Instead, classification by molecular markers is increasingly used to differentiate primary afferent populations, often aligning to specific roles in somatosensation (Usoskin et al., 2015; Arcourt et al., 2017; Huang et al., 2018).

**Figure 1:**
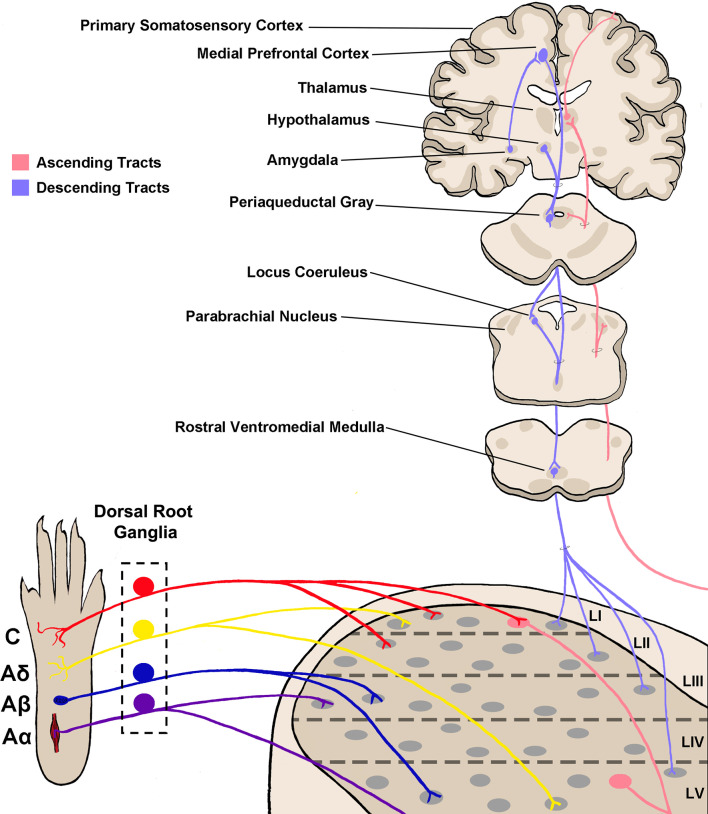
Somatosensory circuitry from the periphery to the brain. Somatosensory information is first transmitted into the spinal dorsal horn by primary afferent neuron fibers, which extend from peripheral tissue into the spinal cord and synapse onto neurons within the dorsal horn. There are four main types of primary afferent fiber, separated by transduction velocity, and each type (Aα, Aβ, Aδ, C) shows some selectivity in somatosensory modality transmitted and synapses into different laminae as shown here (Roberts and Elardo, 1986; McGlone and Reilly, 2010; Koch et al., 2018). This produces selectivity in the modality processed in each lamina of the dorsal horn. Projection neurons within lamina I and V send axons up to the brain *via* ascending tracts including the spinothalamic tract and dorsal column–medial lemniscal tract (not shown in the figure; Willis, 1985; Niu et al., 2013). Briefly, axons decussate at the spinal level, then ascend on the contralateral side towards the thalamus. Some projections terminate within other brainstem regions including the parabrachial nucleus and periaqueductal gray. Descending tracts originate from several brain regions including the medial prefrontal cortex, hypothalamus, and amygdala, and project first to the periaqueductal gray (Gebhart, 2004; Huang et al., 2019). From here, descending projections then synapse within the rostral ventromedial medulla (RVM), and join with locus coeruleus (LC) descending projections. RVM/LC projection targets including the superficial dorsal horn and lamina V (D’Mello and Dickenson, 2008; François et al., 2017).

The dorsal horn is divided into six layers, referred to as laminae, and has long been recognized as a key site for somatosensory processing (Rexed, 1952; Molander et al., 1984). Integrated processing of different somatosensory modalities is achieved through a combination of organizational specificity of primary afferent fiber termination (Figure 1), and through complex circuitry that allows for communication between the laminae of the dorsal horn (Dubner and Ren, 1999; Duan et al., 2014; Bourane et al., 2015; Pagani et al., 2019). The most famous example of this is the gate control theory, which was first theorized by Melzack and Wall (1965) over 50 years ago, and suggests the presence of a network which allows for innocuous stimuli to affect the transmission of noxious stimuli to the brain, rather than these stimuli being separated in distinct networks. Indeed, connections between the deeper dorsal horn layers and lamina I have been defined, which, under pathological pain conditions, allow for a light touch to activate nociceptive projection neurons; a possible neural correlate for allodynia (Takazawa and MacDermott, 2010; Lu et al., 2013; Peirs et al., 2015; Petitjean et al., 2015; Cheng et al., 2017).

Primary afferent fibers may form synapses with excitatory and inhibitory neurons within the spinal cord, and with both interneurons and projection neurons (Lu and Perl, 2003, 2005; Takazawa and MacDermott, 2010). The patterns by which different primary afferent fibers synapse onto which type of dorsal horn neuron, or whether a defined pattern exists, is not definitively known. Part of the reason for this is the high degree of neuronal heterogeneity in the dorsal horn and a lack of information regarding the functional roles of the many types of dorsal horn neurons. Also, no set classification scheme of dorsal horn neurons has been agreed upon. Most early attempts to classify dorsal horn neurons used a classification scheme based on action potential firing patterns or cellular morphology (Prescott and De Koninck, 2002; Punnakkal et al., 2014). However, there can be considerable overlap across these parameters.

More modern classification schemes based on the expression of specific molecular markers have proven more fruitful, with markers such as somatostatin effectively labeling excitatory neurons (Gutierrez-Mecinas et al., 2016, 2019), and markers such as parvalbumin effectively labeling inhibitory neurons (Boyle et al., 2017). It has also recently been found that two classes of neurons, differentiated by expression of substance P or gastrin-releasing peptide, correspond to previously identified morphologically- and electrophysiologically-distinct populations of neurons, namely radial cells and transient central cells, respectively (Grudt and Perl, 2002; Dickie et al., 2019). This provides further evidence that molecular markers can differentiate functionally distinct populations of neurons. However, it should be noted that in some cases molecular markers label both excitatory and inhibitory neurons, as is the case with calretinin (Gutierrez-Mecinas et al., 2016; Boyle et al., 2017), suggesting the need to better understand the heterogeneity of expression of potential molecular markers within this diverse population, in order to find better candidates that align to functionally distinct populations. Recent attempts to use single-cell RNA-sequencing have begun to offer considerable clarity into the complex neuronal heterogeneity in the dorsal horn (Usoskin et al., 2015; Häring et al., 2018; Sathyamurthy et al., 2018; Zeisel et al., 2018), but it is still unknown if these molecularly distinct populations align to different functional populations of neurons within the dorsal horn, representing an open opportunity for research.

The spinal dorsal horn also receives direct descending modulation from several brainstem regions including the rostral ventromedial medulla (RVM) and locus coeruleus (LC; Ren and Dubner, 2002; Gebhart, 2004). These connections serve to modulate the excitability of neurons within the spinal cord, often by decreasing the excitation of cortically-projecting neurons (Figure 1). For example, adrenergic, opioidergic, and cannabinergic signaling from the brainstem can directly inhibit dorsal horn neurons, reduce neurotransmitter release from primary afferents, and ultimately decrease pain behaviors (Ossipov and Gebhart, 1986; Porreca et al., 2002; Huang et al., 2019). Within the dorsal horn, both pre- and postsynaptic sites of action of inhibitory and excitatory neuronal populations have been implicated in descending pain modulation; however, the complete picture of how descending modulation of dorsal horn sensory processing occurs at a systems-level remains unclear (Lau and Vaughan, 2014).

Techniques that allow for manipulation of and recording from specific neuronal populations are ideal for determining how the circuitry of the dorsal horn processes and modulates somatosensory information in normal and pathological conditions. Of increasing utility are recently developed small molecules and genetically-encoded proteins that are activated by light, which allow for either the optical manipulation or detection of activity within spatially or genetically-defined cellular populations; namely optogenetic actuators, and activity sensors such as calcium indicators. The advantages of these tools can be demonstrated by the wide variety of usages they have, both *in vivo* and *in vitro*, from the single cell to population/circuit level; and in the insights that we are deriving from them. They can be used to understand how specifically defined populations of neurons or glia are active during or contribute to the various modalities of somatosensation, how they contribute to crosstalk between these modalities, and how they are affected by descending modulation and/or disorders of sensation including pathological pain conditions.

Here, we will review the current literature on the use of optogenetics for investigation of sensory processing, as well as calcium imaging of both neuronal and glial activity for probing spinal cord circuitry, discuss technologies and limitations, and new advances in both these techniques that could be employed in the future within the spinal dorsal horn.

## Applying Optogenetic Techniques to Probe Spinal Cord Circuitry

### An Overview of Available Optogenetic Tools

Optogenetic tools utilize engineered and artificially introduced ion channels for rapid non-invasive activation or inactivation of specific cellular populations using light (Nagel et al., 2003; Zemelman et al., 2003; Banghart et al., 2004; Boyden et al., 2005; Lima and Miesenböck, 2005; Fenno et al., 2011). These ion channels were first derived from microbial opsins and allow for a wide degree of temporal and spatial sensitivity for manipulation of activity in specific populations of many types of cells, ranging from neurons to HEK cells, and even astrocytes and microglia. They are often referred to as optogenetic actuators, or simply as opsins. The most commonly used excitatory opsin is Channelrhodopsin-2 (ChR2), which upon activation by 488 nm (blue) light allows cations into any cell expressing it (Boyden et al., 2005). Other excitatory optogenetic ion channels have been developed beyond ChR2 that have different or improved functionality, such as oChIEF and Chronos (can be activated at high frequency), Chrimson and C1V1 (activated by a red light), CatCh (permeable to calcium ions), and ChloC (permeable to anions; see Tye and Deisseroth, 2012; Klapoetke et al., 2014, or Lin, 2011 for extensive reviews of different types of opsins and their utilities). However, ChR2 remains the most commonly used excitatory opsin, particularly for interrogation of somatosensation.

The main inhibitory ionotropic opsin is Archerhodopsin-3 (ArchT), which is maximally activated by 530 nm (green) light to allow protons to efflux from the cell, hyperpolarizing the membrane (Han et al., 2011). Halorhodopsin (NpHr) is another commonly used inhibitory opsin, which rather than being an ion channel, functions instead by pumping chloride ions into the cell upon activation with 570 nm (yellow) light (Gradinaru et al., 2008).

To investigate the circuitry of the dorsal horn as well as its function, opsins may be delivered into primary afferent neurons to drive or silence input into the spinal cord, into the brain or brainstem to control descending modulation, or into the spinal cord itself to manipulate the excitability of specific molecularly distinct cellular populations (see Table 1 for an overview of all known usage of opsins for interrogation of spinal dorsal horn somatosensation). Since different opsins are activated by different wavelengths, this allows for the possibility of bidirectional control of excitability in the same preparation (Iyer et al., 2014; Bonin et al., 2016).

**Table 1:** Summary of all publications utilizing optogenetics for interrogation of spinal cord somatosensory circuitry.

		Optical stimulation method			
		*In vitro*	Peripheral	Central stimulation of spinal cord	Central stimulation of descending pathways
Method of opsin expression in tissue	Transgenic	Wang and Zylka (2009) Foster et al. (2015) Honsek et al. (2015) Hachisuka et al. (2016) Bellardita et al. (2017) Uhelski et al. (2017) Pagani et al. (2019) Smith-Edwards et al. (2019) Hachisuka et al. (2020)	Ji et al. (2012) Daou et al. (2013) Bonin and De Koninck (2014) Draxler et al. (2014) Bonin et al. (2016) Christensen et al. (2016) Daou et al. (2016) Stemkowski et al. (2016) Beaudry et al. (2017) Ghitani et al. (2017) Samineni et al. (2017a) Sun et al. (2017) DeBerry et al. (2018) Tashima et al. (2018)	Bonin and De Koninck (2014) Montgomery et al. (2015) Park et al. (2015) Bonin et al. (2016) Bellardita et al. (2017) Lu et al. (2017) Samineni et al. (2017b) Chen et al. (2018) Pagani et al. (2019) Petitjean et al. (2019)	Chen et al. (2018)
	Viral vector	Boada et al. (2014) Yang et al. (2015) Christensen et al. (2016) Hachisuka et al. (2016)	Boada et al. (2014) Iyer et al. (2014) Li et al. (2015) Barik et al. (2018) Mayer et al. (2019)	Iyer et al. (2014) Montgomery et al. (2015) Bonin et al. (2016) Christensen et al. (2016) Nam et al. (2016) François et al. (2017) Mu et al. (2017) Chen et al. (2018) Mondello et al. (2018) Wang et al. (2018)	François et al. (2017) Gao et al. (2019) Huang et al. (2019)

*This table combines all examples of optogenetic interrogation in the dorsal horn on the spinal cord, grouped by the site of stimulation and by the method of opsin delivery*.

### Delivery of Opsins Into Neuronal and Nonneuronal Targets

Through the use of site-specific recombinase technology, opsins can be expressed in subsets of cells by using a specific promoter to selectively express a recombinase such as cre within that subset (Rossant and McMahon, 1999; Nagy, 2000). This technique can be used to drive expression of opsins in a variety of cell types, including neurons, microglia, and astrocytes. However, this does require *a priori* knowledge of the cell type that will be modulated, which is still difficult in the spinal cord where molecularly defined neuronal subtypes are relatively poorly characterized. Recent advances in unbiased molecular screening tools such as single-cell RNA sequencing are beginning to be used to define spinal cord neuronal subtypes and develop atlases of potential target molecular markers for these groupings. This represents a powerful tool for functional characterization, whereby opsins can be expressed within these newly-defined populations to better understand their contributions to dorsal horn circuit function and eventual behavioral output (Abraira et al., 2017; Häring et al., 2018; Sathyamurthy et al., 2018).

Opsins can be delivered into target tissue either through viral transduction or by use of transgenic mouse lines, or through a combination of both technologies (Fenno et al., 2011). Transgenic lines are typically the least invasive method for expression of opsins; however, they are predominantly only available in mice, take time to develop, and are not capable of producing spatially selective expression, save for that given through careful promoter selection (Daou et al., 2013; Liske et al., 2013). Conversely, viral injections can be performed in most animal models and produce spatial selectivity, but do require invasive surgery, and may suffer from sparse expression (Iyer et al., 2014). Along the somatosensory pathways, viral-driven expression of opsins has been achieved with virus injections in the periphery and directly in the sciatic nerve to transduce DRG neurons (Christensen et al., 2016; Wang et al., 2018), by intraperitoneal injection of the virus in neonatal mouse pups to achieve a long-lasting and relatively high degree of DRG transduction (Machida et al., 2013; Vrontou et al., 2013; Bonin et al., 2016; Masuda et al., 2016), by intrathecal injection (Boada et al., 2014), by direct intraspinal injection after laminectomy (Bonin et al., 2016; Mondello et al., 2018) by using a less invasive non-laminectomy approach (Kohro et al., 2015; Petitjean et al., 2019), by injection in the brain to target sensory regions in the brain (Cardin et al., 2009), or through descending pathways (François et al., 2017; Huang et al., 2019). Other approaches, such as intravenous injection of AAVs, can produce low or highly variable transduction efficiencies of the central and peripheral nervous system (Schuster et al., 2014), making them generally less suitable for the optogenetic study of somatosensation.

### Advances in Spinal Cord Optogenetics

#### Peripheral Stimulation

**Figure 2:**
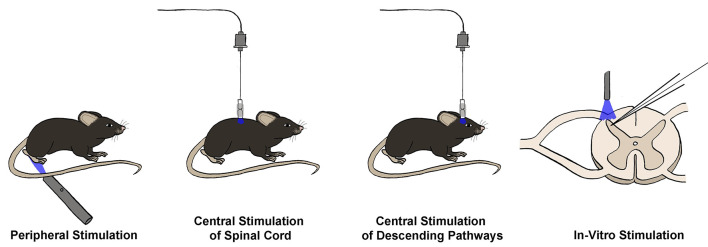
Techniques for optogenetic interrogation of spinal circuitry. Opsins may be stimulated in four main ways to interrogate spinal circuitry. First, opsins can be expressed in primary afferent terminals, and peripheral stimulation can be used to stimulate or silence primary sensory neurons and observe real-time changes in behavioral output *in vivo*. Similarly, opsins can be expressed in neuronal populations within the spinal cord or brain, allowing for *in vivo* central stimulation of either spinal cord neuron populations or neurons involved in descending modulation, respectively. Finally, opsins may be expressed within primary afferents or spinal cord neuron populations, and then stimulation can occur in an *in vitro* slice preparation, often combined with electrophysiological recordings to measure the effect of activation or silencing of a given neuronal population on the excitability of the recorded neuron.

Optogenetic interrogation of spinal cord circuitry can be achieved by using light delivery at several locations, depending on the circuitry that the experimenter is interested in manipulating (Figure 2). Early optogenetic studies began with the insertion of ChR2 into primary afferent neurons, allowing for 488 nm light stimulation of the hind paw to indirectly stimulate circuitry in the spinal cord (Daou et al., 2013). Some of these first sets of experiments targeted ChR2 expression to neurons expressing the sodium channel Nav1.8, which is preferentially found in nociceptive C fiber primary afferent neurons (Agarwal et al., 2004; Daou et al., 2013; Uhelski et al., 2017, but see Shields et al., 2012). Acute stimulation of the hind paw of these animals with 488 nm light led to nocifensive responses such as licking and biting, and enhanced responses to both mechanical and thermal stimuli, suggesting a possible degree of multimodality of these fibers (Daou et al., 2013; Bonin and De Koninck, 2014; Bonin et al., 2016; Daou et al., 2016; DeBerry et al., 2018). Repeated patterned stimulation of the hind paw led to long-lasting mechanical and thermal hyperalgesia, suggestive of potentiation within the spinal cord circuitry. This was further supported by the presence of facilitated dorsal root field potentials after persistent 488 nm light stimulation in a spinal cord explant model (Daou et al., 2013; Bonin and De Koninck, 2014).

A similar technique was used to investigate the effect of activation of two other genetically distinct primary afferent neuron populations on nocifensive behaviors *in vivo*, specifically those that express TrpV1 and those that express MrgD, which are thought to be selectively expressed in peptidergic and nonpeptidergic C fibers, respectively (Zylka et al., 2005; Beaudry et al., 2017). Stimulation of the hind paw with 488 nm light in each of these transgenic mouse lines both resulted in nocifensive responses. However, behaviors were slightly different, suggesting that molecularly distinct populations of C fiber either carry slightly different somatosensory information into the brain or that there is differential processing of information from these fibers within the spinal cord or brain. Conversely, expression of the inhibitory opsin ArchT in primary sensory neurons that express TrpV1 decreases nocifensive responses in a hind paw during 532 nm light stimulation (Li et al., 2015), and activation of eNpHR expressed in C fiber afferents with 570 nm light decreased both thermal and mechanical nocifensive responses, both in naive and nerve-injured animals (Iyer et al., 2014; Bonin et al., 2016).

Expression of ChR2 in the VGLUT3 population of primary afferents, which drives expression within low threshold C fibers, results in 488 nm light-evoked mechanical hypersensitivity in mice with chemotherapy-induced pathological pain but not in naive mice (Seal et al., 2009; Lou et al., 2013; Draxler et al., 2014). This selective pain phenotype adds to evidence that input from these fibers is normally innocuous, and only contributes to nociception in certain pathological pain conditions, possibly through the unmasking of otherwise silent or inhibited synapses within the spinal cord (Li and Zhuo, 1998; Li et al., 1999; Seal et al., 2009; Zhang et al., 2018b). Interestingly, a similar phenomenon was observed when ChR2 was expressed selectively in Thy1+ Aβ fibers, suggesting a similar role (Tashima et al., 2018).

While many of these techniques for peripheral optogenetic stimulation drive opsin expression in molecularly distinct primary sensory neuron populations, some serotypes of AAV have been shown to selectively infect distinct subsets of primary afferent fibers. For example, intrathecal injection of AAV8 encoding ArchT under the ubiquitous CAG promoter was found to selectively induce opsin expression in only Aδ fibers (Boada et al., 2014). Using this technique, Boada et al. (2014) were able to inhibit Aδ fibers *via* activation of ArchT with 532 nm light, which decreased nocifensive responses both in naive animals and after nerve injury, indicating a role for Aδ fibers in both acute and pathological pain conditions. Similarly, Iyer et al. (2014) used an intrasciatic injection of AAV6 encoding ArchT under the CAG promoter to selectively transduce unmyelinated C- fibers, enabling light-induced analgesia.

In summary, peripheral stimulation is the most cost-effective and straightforward method for optogenetic stimulation of spinal cord circuitry and can be accomplished without the need for surgical intervention, depending on the method of opsin expression. Using this technique, the role of different primary afferent populations in various modalities of somatosensation is beginning to be understood and has demonstrated the capacity for light-activated primary afferent stimulation to induce central sensitization within the spinal cord. Unfortunately, peripheral stimulation is primarily suitable for the study of the effect of activation or inactivation of primary sensory afferents on spinal cord functional output in the form of behavioral readouts, and thus central stimulation is required to dissect the circuitry within the spinal cord itself.

#### Central Stimulation

Over the past decade, vast improvements have been made in the utility of optogenetics for manipulating cellular activity. A major advancement has been the capability to stimulate specific cellular populations *in vivo*, using fibreoptic implants for delivery of fixed wavelength light, typically *via* an LED (Aravanis et al., 2007). Initially, fibreoptic implants for the rodent brain suffered from instability and rigidity, necessitating keeping animals in a sedated state during experiments (Adamantidis et al., 2007; Aravanis et al., 2007; Gradinaru et al., 2007). However, new hardware has allowed for awake, *in vivo* optogenetic stimulation of the brain, giving real-time behavioral output (Lima and Miesenböck, 2005; Aravanis et al., 2007; Montgomery et al., 2015).

Researchers have now adapted this fibreoptic technology for direct central stimulation of the spinal cord. Though an initial technique was to use a fibreoptic implant that threads down from the head to the spinal cord (Bonin et al., 2016), a more commonly used approach utilizes a chronic spinal implant in which a short fibreoptic filament and ferrule are cemented into a burr hole in the T13 or L1 vertebre above the L4/L5 spinal segments (Christensen et al., 2016; Pagani et al., 2019). A fibreoptic cable can then be attached to the ceramic ferrule with a ferrule sleeve for stimulation sessions (Figure 2). Using this methodology, one can either directly stimulate primary afferents within the dorsal root ganglia or stimulate spinal cord neurons themselves, with the added benefit of being able to stimulate while the animal is freely moving, which is difficult to achieve with peripheral stimulation.

Central stimulation of primary afferents has been employed by Bonin et al. (2016), who expressed either ArchT or ChR2 in Nav1.8+ primary afferents and delivered light into the spinal cord *via* fiber optic implant. Using this technique, the authors were able to bidirectionally control mechanical nocifensive reflex sensitivity, as measured by Von Frey filaments. Continual excitation of Nav1.8+ afferents led to long-term changes in mechanical sensitivity, measured up to 3 h after stimulation, suggesting the presence of central sensitization.

However, the greatest strength provided by the capability to directly illuminate the spinal cord lies in the capacity to target and either activate or silence specific spinal cord neuronal and glial populations, by expressing opsins in molecularly distinct populations. As technology improves, this will prove to be a very fruitful technique, as many of the newly defined molecularly distinct dorsal horn neuronal populations still have unknown functions in regards to somatosensation in general, and disorders of somatosensation such as pathological pain (Häring et al., 2018). An early study that exemplifies the utility of *in vivo* spinal cord neuron population stimulation using optogenetics found that inhibition of GAD2+ inhibitory spinal cord neurons *via* stimulation of ArchT by 530 nm light produces an immediate increase in mechanical but not thermal sensitivity in otherwise naïve mice (Bonin et al., 2016). This indicates that inhibitory neurons within the spinal cord significantly dampen activation of nociceptive neurons and decrease nociceptive signaling to the brain, giving evidence for how the loss of inhibition could contribute to increased pain sensitivity in pathological pain conditions (Coull et al., 2003, 2005).

A more selective study of interneuron populations found a critical role for the somatostatin positive (SOM+) excitatory neuron population in the circuitry of processing of itch, such that activation of SOM+ neurons *via* ChR2 increased itching behavior *in vivo* (Christensen et al., 2016). Similarly, activation of GRP+ neurons in the spinal cord *via* ChR2 revealed that burst firing within these neurons is required to induce itch-related behaviors *in vivo*, providing evidence that a buildup of GRP neuron activity is required for itch (Pagani et al., 2019).

Finally, a recent study has demonstrated a role for calretinin positive (CR+) neurons in the intersection between innocuous touch perception and nociception. CR+ neurons receive input from multiple sensory afferents responsive to innocuous or noxious stimuli and synapse onto projection neurons within lamina I, providing a direct pathway for elicitation of pain behaviors (Petitjean et al., 2019). Indeed, optogenetic activation of this pathway alone is sufficient to result in mechanical nocifensive responses, without changing thermal sensitivity, suggesting a selective role in mechanical nociception (Petitjean et al., 2019).

It should also be noted that these techniques are not limited to the expression of opsins within neurons, and opsins can also be expressed in nonneuronal cells including astrocytes, for example using the GFAP promoter as done by Nam et al. (2016). By expressing ChR2 in astrocytes in this manner, the authors were able to selectively stimulate spinal astrocytes *in vivo*, finding that astrocyte activation *via* ChR2 leads to pain hypersensitivity, demonstrating a clear link between astrocyte activity and behavioral pain output, possibly through activity-dependent ATP release (Bardoni et al., 2010; Nam et al., 2016).

In the above studies, the light was delivered to the spinal cord *via* a fibreoptic wire attached to a light source external to the animal, such that the wire may interfere with an animal’s native behavior, through the weight or tension of the wire, or through movement restriction (Daou et al., 2013; Towne et al., 2013; Iyer et al., 2014). Therefore, it is of great interest to develop technologies that allow for wireless optogenetic activation, to allow for truly free moving behavior. Wireless light delivery to the spinal cord is still an early technology with only a few successful examples of central modulation of primary afferent terminals in the dorsal horn (Montgomery et al., 2015; Park et al., 2015; Samineni et al., 2017b). However, wireless approaches are thus far unable to consistently deliver sufficient light intensity to activate opsins expressed in the spinal cord parenchyma beneath the myelin and dorsal root entry zone. Continued development of this technology to improve the efficiency of wireless power delivery over larger distances from the power transmitter array and the incorporation of brighter LEDs that can deliver light to deeper laminae will allow this approach to be more broadly applied to the study of spinal somatosensory processing.

A potential complication of utilizing optogenetic tools that must be considered is that there is a risk of exciting not just a desired target cell population, but also neurons or other cell types within the spinal cord itself that also express the chosen molecular target. For example, there are some TRPV1+ neurons within the spinal cord, that could be stimulated during the central stimulation of TRPV1+ primary afferents (Valtschanoff et al., 2001; Roberts et al., 2004; Cristino et al., 2006). Another possibility is that there may be developmental changes in the expression of proteins, resulting in the opsin being expressed in populations of a neuron that previously expressed the molecular marker of interest during an unknown developmental time point, but do not normally express the molecular marker of interest at adult timepoints (Heffner et al., 2012; Song and Palmiter, 2018). Therefore, care should be used both in choosing a genetic marker for a population and in the interpretation of results (Nimmerjahn and Bergles, 2015; Otchy et al., 2015). Another potential strategy to avoid expression in populations with transient, developmental expression would be to only perform viral transfection in adult animals, or to drive recombination only once adulthood has been reached, such as can be achieved with the CreERT2 tamoxifen-dependent recombination system.

Thus far, the results obtained by both central and peripheral stimulation of primary afferent fibers are largely similar, providing encouraging evidence that the location of optical stimulation does not affect the obtained experimental results, and that these potential complications are minimal (Iyer et al., 2014; Nam et al., 2016; François et al., 2017; Samineni et al., 2017a).

Finally, the delivery of light to the spinal cord at an adequate intensity to activate opsins is a major hurdle of spinal cord optogenetics. Unlike the brain, it is not possible to insert a GRIN lens or other optical material directly into the spinal cord parenchyma without severe damage to the spinal cord that can lead to paralysis. Thus, light must be delivered from above the spinal cord, as seen with a vertebral lens or epidural optic fiber implants (Bonin et al., 2016). Also, the spinal cord contains highly myelinated dorsal white matter that can potently scatter light and greatly reduce intensity beyond depths of approximately 100 μm below the surface of the spinal cord (Sekiguchi et al., 2016). The scattering of light by myelin is much greater with short wavelengths (e.g., 488 nm) than long wavelengths of light (e.g., 594 nm), allowing long wavelengths of light to penetrate deeper into the tissue, providing a potential means to mitigate this issue (Zhang et al., 2008; Lin et al., 2013; Chuong et al., 2014; Inoue et al., 2019). Moreover, the degree of light scattering is twice as great when light is delivered from above the spinal cord and perpendicularly to the longitudinal orientation of myelin tracts than if the light is delivered parallel to the tracts (DePaoli et al., 2020). Another complication to light delivery in the spinal cord is that it is much more mobile than the brain and can move up to 50 μm along the rostral-caudal axis during locomotion in mice (Sekiguchi et al., 2016). This necessitates a larger area of illumination to ensure that the light intensity within the spinal region of interest remains sufficiently high to activate opsins as the region moves through the spot of light. Therefore, the intensity of spinally delivered light required to activate opsins expressed by intrinsic spinal cord neurons is higher than that required to activate opsins expressed by sensory afferents, and much higher than that required for opsin activation *via* proximity GRIN lens implantation in the brain.

An alternative method to investigate spinal cord function *in vivo* using optogenetics is through stimulation of the brainstem or brain to determine the effects of descending modulation on behavioral output. The spinal cord receives a great deal of descending input *via* tracts from many key brain and brainstem areas, including the prefrontal cortex, hypothalamus, amygdala, and periaqueductal gray (PAG; Hopkins and Holstege, 1978; Gebhart, 2004; François et al., 2017; Huang et al., 2019). These inputs converge at the LC and RVM, which synapse directly into the spinal cord (Proudfit and Clark, 1991; D’Mello and Dickenson, 2008; François et al., 2017). Input from the LC and RVM is thought to modulate excitability through both facilitation and inhibition, depending on the neuronal populations involved. To dissect out these roles, opsins can be selectively expressed in the spinally-projecting brainstem or brain neurons. Using this technique, a novel basolateral amygdala—medial prefrontal cortex—PAG—spinal cord pathway has been identified, that when optogenetically activated, inhibits nocifensive behaviors in mice with a pathological pain condition (Huang et al., 2019). Conversely, rostral ventromedial medulla GABAergic interneurons appear to function to increase nocifensive behaviors in mice, with activation of ChR2 increasing nocifensive behaviors, and activation of NpHR decreasing nocifensive behaviors *in vivo* (François et al., 2017).

#### *In vitro* Spinal Cord Optogenetics

In addition to observing the effect of stimulation or inhibition of neuronal populations on behavioral output *in vivo*, valuable information can be gained from combining optogenetic stimulation with the recording of the electrical activity of dorsal horn neurons, either through field or patch-clamp recordings. While a few labs have successfully been able to perform electrophysiology from the spinal cord *in vivo*, albeit under anesthesia (Light and Willcockson, 1999; Sokal and Chapman, 2003; Urch and Dickenson, 2003; Keller et al., 2007), for most practical applications either a semi-intact preparation (Hachisuka et al., 2016), spinal cord explant (Bonin and De Koninck, 2014), or spinal cord slices are used (Figure 2; Daou et al., 2013; Christensen et al., 2016; Pagani et al., 2019).

Though the semi-intact and explant preparations allow for dorsal roots to remain intact, in some cases dorsal roots are removed during the preparation of spinal cord slices. However, even with dorsal roots removed, optogenetic excitation of central terminals of primary afferent neurons is sufficient to produce postsynaptic responses in spinal cord neurons (Wang and Zylka, 2009; Foster et al., 2015). For an investigation of primary afferent-evoked responses within the spinal cord, optogenetic stimulation provides the benefit of selectively stimulating a genetically distinct subset of primary afferents, as compared to electrical stimulation (Tashima et al., 2018; Kubota et al., 2019). This provides unparalleled specificity to define circuitry within the spinal cord. For example, Wang and Zylka (2009) expressed ChR2 in MrgD+ primary afferents that are nonpeptidergic multimodal C fiber primary afferents (Rau et al., 2009) and then recorded from a large number of substantia gelatinosa neurons. The authors found that 50% of all recorded substantia gelatinosa neurons receive monosynaptic input from MrgD+ afferents and that all morphologically defined classes of substantia gelatinosa neurons receive these inputs, as evidenced by light-evoked excitatory postsynaptic potentials (EPSCs).

As described earlier, opsins can also be expressed directly within molecularly distinct spinal cord populations, for example within inhibitory VGAT+ or GAD2+ neurons (Foster et al., 2015; Bonin et al., 2016). In a spinal cord slice preparation, this allows for dissection of circuitry through activation or suppression of neuronal populations during patch-clamp electrophysiology, or possibly in combination with calcium imaging. The former technique has been employed to determine the connections from pruritogenic primary afferents to the spinal cord, which synapse onto GRP+ neurons, then on to GRP receptor-containing neurons, and finally onto glutamatergic parabrachial nucleus-projecting neurons (Mu et al., 2017; Pagani et al., 2019), elucidating a full circuit from the periphery to the brain for the flow of itch information. This technique has also been employed in conjunction with *in vivo* optogenetics extensively, to determine presynaptic and postsynaptic connections between populations like CR+ and SOM+ neurons (Daou et al., 2013; Bonin and De Koninck, 2014; Christensen et al., 2016; François et al., 2017; Petitjean et al., 2019).

Overall, these insights are beginning to unravel the complex circuitry of the spinal cord and allow for dissection of the contributions of specific neuronal populations to somatosensory processing, and especially that involved in acute and pathological pain conditions. In the future, it will also be of interest to investigate the intersection between innocuous touch and pain, and how these connections have been hypothesized to be altered in pathological pain conditions.

## Fluorescence Imaging for Measurement of Spinal Dorsal Horn Neuronal and Glial Activity

### Tools for Fluorescence Imaging of Neuronal and Glial Activity

Historically, the only way to measure the activity of individual neurons has been through electrophysiology. However, fluorophores have now been engineered to undergo conformational changes leading to increased fluorescence in the presence of biologically relevant stimuli, such as a change in voltage or calcium concentration. This allows for non-invasive monitoring of relative cellular activity with single-neuron precision; with the additional benefit of the capability to record from up to hundreds of neurons simultaneously. These powerful tools give insight into the activity of ensembles of neurons, to better understand their function within complex circuitry such as the spinal cord.

The most common activity-sensing fluorophores are calcium and voltage indicators, which upon binding of calcium ions or a change in voltage across the membrane, respectively, proportionally increase fluorescence intensity within a set linear dynamic range (Grynkiewicz et al., 1985; Minta et al., 1989; Tsien, 1989; Chanda et al., 2005; Fromherz et al., 2008; Bradley et al., 2009). These fluorophores may either be chemical indicators or genetically-encoded proteins; and the degree of increase in fluorescence intensity upon binding, the wavelength of light required to cause this conformational change, and the sensitivity of fluorophores differs widely, creating a vast toolbox of possible indicators for any desired application (see Grienberger and Konnerth, 2012 for the table of common chemical indicators, see Lin and Schnitzer, 2016 for a table of common genetically-encoded calcium indicators).

It should be noted that fluorophores have also been created for imaging of other molecules relevant to cellular activity, such as the fluorescent chemical indicator MQAE and genetically-encoded protein Chlomeleon for chloride measurement (Arosio and Ratto, 2014), iGluSnFr for fluorescent measurement of glutamate (Marvin et al., 2013, 2018), and Epac-based FRET sensors for cAMP measurement (Ponsioen et al., 2004; Klarenbeek and Jalink, 2014). Additionally, fluorescent false neurotransmitters have been developed to study the release and recycling of neurotransmitters such as dopamine (Gubernator et al., 2009). However, imaging data obtained using these molecules are not as strongly associated with the cellular activity as calcium or voltage imaging and thus will not be discussed further in this review.

The best proxy for electrophysiology currently available is the imaging of voltage-sensitive fluorophores, which are capable of detecting both action potentials and subthreshold changes in voltage (Xu et al., 2017). Unfortunately, most voltage indicators suffer from poor signal-to-noise ratios (SNRs) and require a high intensity of light for stimulation, which is particularly difficult to overcome in the spinal cord. Part of the necessity for higher intensity light is because voltage indicators are only expressed on the cell membrane, which accounts for only a small portion of the overall volume of a neuron. Newer indicators such as Voltron (Abdelfattah et al., 2019), QuasAR2 (Hochbaum et al., 2014), and ASAP3 (Villette et al., 2019) provide better SNR and show greater promise for use in living tissue. All three of these newer generation voltage indicators have successfully been expressed *in vivo* in the mouse cortex, with ASAP3 and Voltron capable of discerning single action potentials on the millisecond timescale (Lou et al., 2016; Bando et al., 2019). Unfortunately, there has only been one publication performing voltage imaging within the spinal cord, using the voltage-sensitive dye Di-4-ANEPPS, which lacked single-neuron resolution (Mizuno et al., 2019), leaving voltage imaging in the spinal cord an open avenue for innovation. Instead, most researchers use calcium imaging as a proxy to estimate neuronal activity.

### Considerations When Using Calcium Imaging as a Proxy for Neuronal Activity

Calcium imaging takes advantage of the large, rapid flow of calcium into neurons as a result of voltage-gated calcium channel (VGCC) opening during action potential firing (Grynkiewicz et al., 1985; Minta et al., 1989; Tsien, 1989; Hahn et al., 1990). Because calcium concentration within neurons rises from the nanomolar to the micromolar range after firing of a single action potential, and because calcium indicators can be expressed within the entire cytosol rather than just on the cellular membrane, calcium imaging has typically provided an intrinsically higher SNR than voltage imaging (Grienberger and Konnerth, 2012; Kulkarni and Miller, 2017). As a result, calcium imaging has become the gold standard for fluorescence imaging of both neuronal and glial activity.

**Figure 3:**
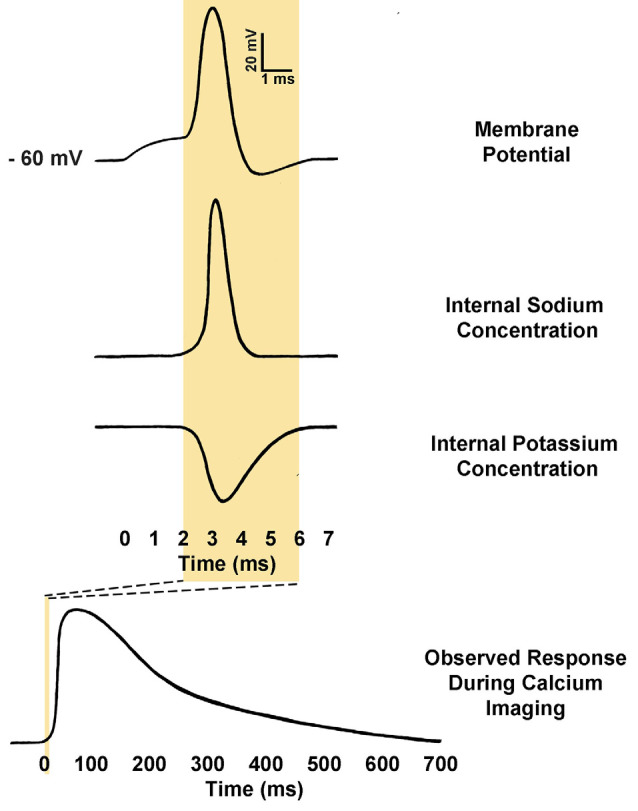
Comparison of action potential kinetics and calcium imaging responses. Calcium indicators are used to measure relative calcium concentration, and this is often used as a proxy for action potential firing. Action potentials consist of an initial depolarization to action potential threshold, which opens voltage-gated sodium channels, causing rapid depolarization of membrane potential. The membrane potential is then rapidly repolarized by the opening of voltage-gated potassium channels. As a result, a typical action potential may only last from 2 to 5 ms. Conversely, observed calcium responses within the somatic cytosol of neurons during action potential firing may require anywhere from 500 to 1000 ms to return to baseline fluorescence (Rahmati et al., 2016). The kinetics of the response is determined in part by the calcium buffering of the neuron itself as well as the affinity and concentration of the calcium indicator present (Borst and Abarbanel, 2007; Hires et al., 2008).

Using calcium imaging as an indirect readout of action potential firing in a neuron requires consideration of several factors regarding the time course of calcium signals. First, the kinetics of calcium concentration changes within a neuron during action potential firing differ widely from the kinetics of the action potential itself. Whereas an action potential occurs on a millisecond time scale—with voltage rapidly depolarizing *via* sodium entry and subsequently hyperpolarizing *via* potassium exodus from the neuron—calcium responses to action potential firing are both much slower to rise and much slower to return to basal concentration after action potential firing (Figure 3; Yang and Wang, 2006; Bean, 2007). The rise in calcium is delayed because calcium entry is typically through activation of VGCCs, which will only open once a threshold depolarization has been reached, and which have slower activation kinetics than voltage-gated sodium channels (Carbone and Swandulla, 1989; Bean, 2007). The voltage threshold for activation can vary depending on VGCC subtype, but typically would be near the end of the rising phase of the action potential itself; as is the case for the highly abundant L-type VGCCs, which have an activation threshold around −20 to 0 mV (Catterall, 2011; Striessnig et al., 2014). Slower rise times in intracellular calcium concentration will also be observed depending on the affinity and concentration of the calcium indicator. This is because every calcium indicator is also a calcium buffer, and increased buffering will subsequently increase the duration for molecules of a calcium indicator to bind to all available calcium ions, and reach peak fluorescence (Borst and Abarbanel, 2007; Hires et al., 2008).

In addition to slower rise times as compared to the native action potential they are reporting on, calcium indicators are also much slower to decay and return to basal calcium concentration. Typically, the calcium response to a single action potential will decay within a neuron over hundreds of milliseconds, or perhaps even seconds with high-affinity indicators (Rahmati et al., 2016). This slow decay time becomes especially problematic with high-frequency firing, where it can become impossible to resolve the peaks of individual action potentials (Smetters et al., 1999). The capacity of a given calcium indicator to accurately report on the number of action potentials fired in the sequence depends on a multitude of factors, including the calcium indicator’s affinity for calcium, the native calcium buffering present within the neuron, and extrusion rates present within that neuronal cell type (Mank and Griesbeck, 2008; Paredes et al., 2008; Tian et al., 2009; Akerboom et al., 2013; Podor et al., 2015). A final technical consideration is the frequency of image collection. For example, if one is measuring action potentials at 100 Hz, the frame sampling rate must at minimum exceed 200 Hz to resolve individual peaks, as per the Nyquist theorem. An inability to resolve individual peaks can also be an issue even at lower firing frequencies if there is a large train of action potentials, as calcium can begin to accumulate quickly within the intracellular space and exceed the linear range of the given calcium indicator (Hires et al., 2008). Thus, one must put careful consideration into the choice of calcium indicator and pick one optimal for their research parameters.

### Choosing a Calcium Indicator

Three main considerations determine the type of calcium indicator to employ in an experimental paradigm: the wavelength of activation, the method of delivery into the tissue, and the affinity of the indicator. Indicators activated by blue light still have the best signal to noise ratio (SNR) properties and should be considered first (Dana et al., 2019). However, these indicators may not be possible for certain applications; for example, when combining calcium imaging with optogenetics, a red-shifted calcium indicator would be required to avoid activation of ChR2 during imaging of the calcium-sensitive fluorophore. A red-shifted calcium indicator may also be preferable for imaging at depths below 100 μm, whenever two-photon imaging is not possible, as longer wavelengths are capable of penetrating deeper into tissue (Sanderson et al., 2014).

**Figure 4:**
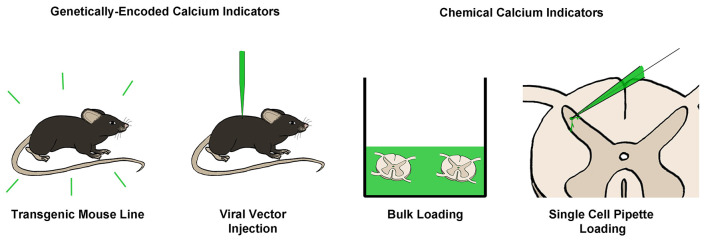
Common techniques for loading of calcium indicators. Calcium indicators can broadly be categorized into genetically-encoded (GECIs) or chemical calcium indicators. GECIs can be introduced *via* transgenic mouse lines or through the injection of a viral vector containing a GECI into the target tissue. Chemical calcium indicators can be introduced in several ways, however, the most common is through bulk loading with an AM-ester bound indicator, or *via* single-cell pipette loading. Both of these techniques can be performed *in vitro* or *in vivo*.

Next, one must consider the method of delivery of the calcium indicator into tissue (Figure 4). Transgenic mouse lines and viral injections of genetically-encoded calcium indicators (GECIs) are becoming increasingly common, especially with the significant advances in the caliber of GECIs in recent years; particularly the GCaMP6 line of GECIs, which have SNRs similar to the best chemical calcium indicators (Chen et al., 2013b). The new jGCaMP7 line promises even better SNR, exceeding the capability of traditional chemical indicators like Fura-2 or OGB-1 (Dana et al., 2019). For red-shifted GECIs, jrGECO1a (Dana et al., 2016) or KGECO1 (Shen et al., 2018) could be employed.

For some applications, chemical calcium indicators may still be preferred. These include experiments involving animals in which transgenic lines are less common or not possible, such as rats or primates, or situations in which a viral injection may not be possible. For these types of experiments, bulk loading of a membrane-permeable AM ester-attached calcium indicator may be employed. In these experiments, the indicator will load into many neurons simultaneously and remain trapped within the neuron for hours afterward upon cleaving of the AM ester by native esterases. One other method that may be employed is single-cell loading *via* a patch pipette, which is often preferred for detailed investigation of dendritic arbor as it provides superior SNR because of reduced background (Helmchen et al., 1999; Grienberger and Konnerth, 2012).

Finally, as described above, the affinity of the chosen indicator will determine the kinetics of the measured calcium response. For experiments where single action potentials need to be resolved, a high-affinity indicator should be used, with the caveat that the decay of the response will be longer. However, for experiments where a train of action potentials need to be resolved—especially with a high-frequency train—a lower affinity indicator should be used to improve the kinetics of the response, and for the indicator to be performing within its linear dynamic range for the duration of the stimulus (Pologruto et al., 2004; Hollingworth et al., 2009).

### Advances in Spinal Cord Fluorescence Imaging of Activity

#### Calcium Imaging of Spinal Cord Neurons *in vitro*

Calcium imaging has thus far not been employed as widely within the spinal dorsal horn as in the brain, largely because of technical limitations that have hindered its utility. This is especially true for *in vivo* studies, of which only a handful have been conducted in the spinal cord. One of the first calcium imaging experiments used epifluorescence microscopy to show that overall basal calcium indicator fluorescence was higher on the ipsilateral side of a transverse spinal cord slice from an animal with the chronic constriction injury model of pathological pain, suggestive of either increased resting calcium concentration or increased neuronal activity in the absence of external stimulation within the ipsilateral dorsal horn (Kawamata and Omote, 1996). Using a similar technique, it was also found that the calcium response to dorsal root stimulation on the superficial dorsal horn ipsilateral to nerve injury was larger, indicative of increased neuronal activity for the same stimulation, mirroring the hyperalgesia present in pathological pain conditions (Luo et al., 2008).

Since these first studies, improvements both in calcium indicators and in microscope technology now allow for single neuron and single event resolution. This provides the capacity to use calcium imaging for the measurement of discrete calcium signals within dorsal horn neurons, such as those due to action potential firing, and neuronal responses to an applied stimulus like capsaicin (Merighi et al., 2008), or electrical stimulation (Kim et al., 2015; see Table 2 for a full list of all known applications of calcium imaging within the spinal dorsal horn).

**Table 2:** Summary of all publications utilizing calcium imaging for interrogation of spinal cord somatosensory circuitry.

		Imaging method		
		Epifluorescence	1-photon	2-photon
Method of delivery of the calcium-sensitive indicator	Transgenic	Bellardita et al. (2017)	Sun et al. (2017)	Tang et al. (2015)* Zhang et al. (2018a)* Olivares-Moreno et al. (2019)
	Viral Vector	Simonetti et al. (2013) Aresh et al. (2017) Freitag et al. (2019)	Nishida et al., 2014* Sekiguchi et al., 2016*	Yoshihara et al. (2018)* Sekiguchi et al. (2016)* Chen et al. (2018)*
	Bulk Loading	Kyrozis et al. (1995) Kawamata and Omote (1996) Coull et al. (2003) Tsuzuki et al. (2004) Cordero-Erausquin et al. (2005) Coull et al. (2005) Ruscheweyh and Sandkuhler (2005) Shutov et al. (2006) Luo et al. (2008) Schoffnegger et al. (2008) Miyano et al. (2010) Flynn et al. (2011) Doolen et al. (2012) Corder et al. (2013) Petitjean et al. (2014) Baba et al. (2016) Gao et al. (2016) Doolen et al. (2017) Potter et al. (2018) Skorput et al. (2018) Taylor et al. (2019)	Gustafson-Vickers et al. (2008) Merighi et al. (2008) Bardoni et al. (2010) Lu et al. (2018)	Wilson et al. (2007) Johannssen and Helmchen (2010)* Laffray et al. (2011) Cirillo et al. (2012)* Cirillo et al. (2015)* Ran et al. (2016)* Skorput et al. (2018)
	Single Cell Pipette Loading	Isaev et al. (2000) Ikeda et al. (2003) Heinke et al. (2004) Kopach et al. (2011) Yan et al. (2014) Kim et al. (2015) Yan et al. (2019)	Drdla et al. (2009)*	Ikeda et al. (2006)* Drdla et al. (2009)

This table amalgamates all examples of calcium imaging in the spinal dorsal horn, with references grouped by imaging technique and by the method of delivery of the calcium-sensitive indicator. *Beside a citation indicates that some or all of the experiments were performed in vivo.

Calcium imaging can also be used to investigate the relative function of calcium-permeable ion channels, including calcium-permeable AMPA receptors, NMDA receptors, and VGCCs within the spinal dorsal horn, especially concerning the spatial distribution of calcium. Recent investigations have utilized calcium imaging to study how calcium enters into neurons during activity. Using a bulk-loaded calcium indicator and high-resolution epifluorescence imaging, Doolen et al. (2012) were able to measure spontaneous action potentials as calcium responses in single dorsal horn neurons. They subsequently developed a technique to use calcium imaging to quantify relative glutamate receptor function by measuring glutamate-evoked calcium events in single superficial dorsal horn neurons from transverse slices. Using this technique, they found that calcium events to the same glutamate stimulus were larger on the side ipsilateral to nerve injury, indicative of either upregulation of glutamate receptor density or an increase in glutamate receptor conductance. Later, they found that these calcium events could be further boosted in a pain model by blocking opioid receptors (Corder et al., 2013). A similar study by Skorput et al. (2018) found that glutamate-evoked calcium responses in superficial dorsal horn neurons were significantly higher upon application of a peptide derived from VGF nerve growth factor, indicating that VGF is capable of potentiating glutamatergic signaling in the spinal cord.

In addition to measuring the activity of calcium-permeable glutamate receptors, calcium imaging has also been used to define relative contributions of VGCC subtypes to activity-evoked calcium responses. The first study to investigate this introduced the calcium indicator Fura-2 into spinal cord lamina I neurons *via* a patch pipette and measured calcium responses within the soma in response to current injection-evoked 40 Hz action potential firing (Ikeda et al., 2003). However, the authors were not able to identify discrete intracellular action potential-induced calcium responses due to the firing frequency and properties of Fura-2, which has a high calcium-binding affinity and reaches saturation below a calcium concentration of 1 μM (Paredes et al., 2008). Rather, it was observed that the total calcium response to the stimulation protocol was larger in projection neurons, largely due to the expression of additional T-type VGCC channels in these neurons that enhanced depolarization and increased total calcium influx component. This finding was then expanded to determine the relative contributions of various VGCCs to the somatic calcium response induced by the burst of action potentials. In unlabeled lamina-I neurons, of which the majority are likely interneurons, the major contributor to this calcium response was L-type VGCCs with significant T-type and N-type contributions (Heinke et al., 2004).

A key strength of calcium imaging is the ability to investigate the spatial dynamics of calcium with subcellular resolution. Within the brain, calcium imaging has been extensively used to investigate postsynaptic calcium entry (Harvey et al., 2008; Lee et al., 2009; Zito et al., 2009), dendritic calcium signaling (Spruston et al., 1995; Gulledge et al., 2005; Larkum et al., 2009; Harnett et al., 2012), and nuclear calcium dynamics (Eder and Bading, 2007; Bengtson and Bading, 2012) using two-photon calcium imaging. In superficial dorsal horn neurons, calcium imaging has been used to observe calcium entering into the nuclei of neurons during repetitive primary afferent stimulation (Simonetti et al., 2013). Inhibiting nuclear calcium signaling through the expression of CaMBP4, which binds calcium-bound calmodulin and thereby prevents calmodulin-dependent downstream signaling cascades, significantly reduced hypersensitivity in mice exposed to a Complete Freund’s Adjuvant model of chronic inflammatory pain, suggesting that the development of pathological pain requires calcium to enter into the nucleus to initiate downstream signaling cascades. Thus far, there have been no other studies investigating the subcellular calcium dynamics of dorsal horn spinal cord neurons, and this represents an open field of study.

Together, these studies demonstrate the utility of calcium imaging for more than measuring neuronal activity, but also for the visualization and measurement of calcium itself within cells, including both neurons and glia. As demonstrated above, subcellular calcium imaging can provide important information on calcium’s role as a critical second messenger for initiating downstream signaling cascades that lead to changes in protein expression and cellular function. Another emerging opportunity involves combining optogenetics to drive specific neuronal populations with calcium imaging to monitor resultant cellular activity within the spinal cord. This combination would be especially helpful when measuring population ensemble activity (Ruscheweyh and Sandkuhler, 2005), or when animals are freely moving and electrophysiological recordings would not be possible.

#### Challenges to Achieving Freely Behaving *in vivo* Calcium Imaging in the Spinal Cord

Effective calcium imaging requires precise spatial and temporal resolution, which can be difficult to achieve *in vivo* in the spinal cord, and considerably more difficult to achieve in a freely moving animal. Several attempts to achieve this goal have utilized miniscope or endoscope systems (Johannssen and Helmchen, 2010; Sekiguchi et al., 2016). These types of systems allow for free movement in the mouse, restricted only by a cable leading to a computer, and the connected miniscope or endoscope itself, which often weighs only a few grams (Yang and Yuste, 2017; Zhang et al., 2018b). However, this type of system suffers from lack of tissue penetration and decreased resolution as compared to imaging under a two-photon microscope. This resolution is likely not sufficient for the monitoring of subcellular events, and in the case of endoscope systems, is often insufficient to differentiate and record from individual neurons. Additionally, as described earlier, light scattering in the spinal cord due to myelination can prevent epifluorescent and single-photon imaging from detecting fluorescent cell bodies deeper than the outer part of lamina II (Sekiguchi et al., 2016), making two-photon imaging a necessity for investigating deeper laminae. Two-photon microscopy also benefits from superior spatial resolution, reduced phototoxicity, and can be used to achieve subcellular resolution of calcium dynamics (Dana et al., 2019).

Unfortunately, with greater spatial resolution comes the need for stability in the imaging field. Therefore, with current technology, almost all *in vivo* two-photon calcium imaging in the brain has been performed on head-fixed animals (Chen et al., 2013a; Leinweber et al., 2014; Yang and Yuste, 2017; but see Helmchen et al., 2013 and Zong et al., 2017). Translating these techniques to the spinal cord has been met with middling success, partially because it is significantly more difficult to fix the spinal cord in place for imaging, with breathing and movement producing significant imaging artifacts that are difficult to compensate for while allowing for enough spinal flexibility for free movement.

This fine yet necessary balance of movement and fixation that must be achieved for *in vivo* two-photon calcium imaging represents such an obstacle that only one research group has thus far successfully achieved this, by placing a mouse mounted on a spherical treadmill under a two-photon microscope (Sekiguchi et al., 2016). For this study, the authors tested several methods of head and vertebral restraint to optimize imaging conditions, finding that a dual head and vertebral restraint provided a maximum reduction in motion artifacts while maintaining locomotor activity, and demonstrating the feasibility of the technique.

The authors then performed two-photon imaging of axonal arbor, as well as calcium imaging of astrocytic processes, and importantly found that pinch-evoked astrocytic calcium responses were depressed under anesthesia (Sekiguchi et al., 2016). This finding provides crucial evidence that within the spinal cord, there is a necessity for further refining techniques to allow for two-photon imaging that does not require anesthesia. The potential impact of anesthesia is of special interest in the context of another recent study that expressed GCaMP6m in spinal cord astrocytes using a viral vector injection and performed *in vivo* two-photon calcium imaging under anesthesia. Here, the authors found no astrocytic calcium responses to pinch or brush in naive animals but surprisingly found astrocytic calcium responses to these innocuous stimuli after animals received a formalin injection (Yoshihara et al., 2018). It is therefore, possible that these findings could be even more pronounced in awake animals.

Additionally, through the implantation of a spinal imaging chamber attached to a miniscope, it has been found that anesthesia decreases spontaneous calcium events in superficial dorsal horn neurons from 0.52 Hz to 0.08 Hz, representing a marked depression in circuit activity (Sekiguchi et al., 2016). This calcium event rate measured using calcium imaging is remarkably close to the average action potential firing rate measured using *in vivo* extracellular single-unit recordings of superficial dorsal horn neurons under anesthesia (0.05 Hz; Keller et al., 2007), and provides further support that a system of activity measurement not requiring anesthesia represents a fundamental advance for decoding and understanding the circuitry of the spinal cord.

However, due to the many difficulties outlined above, the majority of *in vivo* two-photon imaging experiments in the spinal cord have thus far been performed under anesthesia (Ikeda et al., 2006; Davalos et al., 2008; Drdla et al., 2009; Laffray et al., 2011; Cirillo et al., 2012; Farrar et al., 2012; Fenrich et al., 2013; Nishida et al., 2014; Cirillo et al., 2015; Ran et al., 2016; Chen et al., 2018; Wang et al., 2018). Several early studies focused on technique development to determine the best approaches for exposing spinal cord tissue to the microscope and for limiting breathing artifacts. One of the first such studies expressed fluorescent molecules in the axons, microglia, and blood vessels of mouse spinal cord to monitor structural changes over time, and developed a spinal stabilization device that provided a high degree of stability for imaging (Davalos et al., 2008). This method was utilized to perform the first two-photon *in vivo* calcium imaging within the superficial dorsal horn in which OGB-1 AM was injected into the dorsal horn and bulk loaded into superficial dorsal horn cells (Johannssen and Helmchen, 2010). In this study, the authors found many cells to have spontaneous calcium responses, as well as evoked calcium responses to electrical and mechanical stimulation of the hind paw.

*In vivo* two-photon calcium imaging has also been used to monitor long-term potentiation (LTP) in superficial dorsal horn neurons, using calcium responses as a proxy for neuronal activity. Interestingly, the authors found that calcium responses were slower to rise and fall in PAG-projecting spinal cord neurons, as opposed to PBN-projecting neurons, and that this slower, longer calcium response was correlated with a greater degree of LTP in that neuron (Ikeda et al., 2006).

Unfortunately, all of these studies required a head-support, spinal column, and tail-clamping, as well as deep anesthesia. As a result, these imaging sessions were terminal and precluded long term imaging of cellular changes accompanying chronification of hyperalgesia. Newer techniques have been developed in which a glass chronic spinal imaging window is implanted, which allows for chronic imaging at multiple time points over the course of months (Farrar et al., 2012; Fenrich et al., 2013; Chen et al., 2018). An added benefit of a chronic spinal imaging window is that after implantation, and at later time points, the inflammatory response is minimal, giving better insight into the native function of the spinal cord (Fenrich et al., 2013).

Three recent studies have investigated *in vivo* dorsal horn neuron calcium responses to several different modalities of hind paw stimuli, including pinch, brush, cold, and heat (Nishida et al., 2014; Ran et al., 2016; Chen et al., 2018). These studies have found significant overlap in the modalities that can activate individual neurons, as well as grading in both the percentage of neurons active and the amplitude of the ultimate measured calcium response. Notably, one study found that stimulating the anterior cingulate cortex resulted in potentiation of the calcium response in over 50% of pinch-responding neurons in the dorsal horn, indicating rapid descending modulation of spinal cord neuron excitability (Chen et al., 2018). These initial results will be incredibly interesting to investigate further through combination with optogenetic stimulation, or with the labeling of genetically distinct neuronal populations, to begin to delineate which spinal cord cell types contribute to the processing of different sensory modalities.

Although not strictly within the spinal cord, two complementary studies to the ones above were recently performed for primary afferent neuron cell bodies within the dorsal root ganglia (DRG), measuring *in vivo* calcium responses in DRG cell bodies to various stimuli (Chisholm et al., 2018; Wang et al., 2018). In these studies, GCaMP6 was introduced into DRG cell bodies through injection of the AAV9 or AAV8 viral vectors, respectively, under a general CAG promoter. Various thermal and mechanical stimuli were presented to the hind paw while the mice were under anesthesia and spinal restraint. By studying the responses of a large population of cells to multiple stimuli, both studies concluded that many primary afferent neurons were polymodal, responding both to thermal and noxious mechanical stimulation. Wang et al. additionally found that heat and cold are differentially encoded by DRG activity, such that increasing heat produced a graded response in a subset of heat-sensitive neurons, while increasingly cold temperatures activate distinct subsets of cells to reveal a population level encoding. In the future, it is hopeful that these types of study will also be performed within the spinal cord, and without the necessity for anesthesia.

In summary, fluorescence imaging is a powerful tool for the monitoring of cellular activity. Although several considerations must be taken into account to most effectively employ this technology, imaging remains an ideal way to monitor the activity of hundreds of neurons or glial cells simultaneously at the overall network level or in genetically defined subpopulations, providing invaluable information about neuronal circuitry and function. Calcium imaging can effectively be adapted for a wide variety of paradigms, owing to the multitude of ways that calcium indicators can be introduced into cells, and the many experimental paradigms in which it can be employed; ranging from single-cell patch pipette loading to two-photon *in vivo* imaging of genetically-encoded calcium indicators. While there are fewer examples of imaging with voltage-sensitive indicators, likely owing to the relatively new development of indicators with sufficient SNR for use in tissue, advances in imaging technology and novel voltage indicator development have paved the way for wider use of this imaging modality.

## Summary and Outlook

Optical approaches allow for less invasive and highly selective manipulation and measurement of cellular activity. Given the complexity of the circuitry involved in the processing of somatosensory information, these tools promise to aid in unraveling how somatosensory information is encoded and integrated within the spinal cord, and how these processes change in conditions such as pathological pain.

Optogenetic tools such as ChR2 and ArchT have been effectively utilized to rapidly and non-invasively activate and inactivate specific neuronal populations, respectively. Molecular genetic approaches have allowed for directed, tissue-specific opsin expression with the cre-lox system, and surgical advances have paved the way for precise, stereotaxic injections for virally-mediated transduction. The ability to transgenically or virally introduce both excitatory and inhibitory opsins to the same population of neurons has opened new possibilities to study the role of discrete spinal cord neuronal populations with remarkable resolution. Using these techniques, optogenetics has been used in a multitude of studies, from defining the contributions of various distinct primary afferent fibers in somatosensation to single-neuron interrogation of spinal dorsal horn circuitry.

As the gold standard for fluorescence imaging of cellular activity, calcium imaging provides the capacity to detect discrete events within single cells, while simultaneously recording from ensembles of neurons in the spinal cord. This is especially powerful when utilizing genetically-encoded calcium indicators that can be driven to molecularly distinct populations of neurons or glia, and can be used to answer many outstanding questions in the field, including the possibility of neuronal synchrony, and how activity in populations of spinal cord neurons and glia determine freely moving behaviors in real-time.

Given the high diversity of calcium indicators, we have presented three major considerations in choosing a calcium indicator: the desired activation wavelength, tissue delivery technique, and indicator affinity. By considering these variables, calcium imaging experiments can be utilized optimally within the spinal cord. Technological advances in both calcium indicators and microscopy now allow for single-cell, and subcellular resolution with an *in vivo* approach, but major challenges concerning microscope stability and anesthetic conditions remain. Nonetheless, the utility of calcium imaging as a measure of cellular activity, and the relative degree of non-invasiveness compared to electrophysiology, makes this technique invaluable for elucidating spinal cord circuitry function at both single-cell and network-level resolution.

The determination of brain circuitry in behavior is moving towards all-optical interrogation, which combines the comprehensive readout of cellular activity *via* calcium imaging with the spatial and temporal precision of neuronal activation *via* optogenetics. While the technical difficulties are substantial, and especially so within the spinal cord, this approach could provide the opportunity to define somatosensory circuitry with unprecedented detail. Through combining expression of blue-shifted and red-shifted indicators and opsins, such as the combination of ChR2 with jrGECO1a or Chrimson with GCaMP7, experimenters will be able to activate or silence one population of cells at one wavelength and measure the changes in the activity of another population, all *in vivo*, allowing for simultaneous readout of behavior (Rickgauer et al., 2014; Packer et al., 2015; Stamatakis et al., 2018; Zhang et al., 2018c). Together, these tools provide the future possibility for closed-loop interrogation of spinal cord circuitry and function *in vivo*, to understand how somatosensory information is processed in this incredibly complex network.

## Author Contributions

All authors planned the structure of the manuscript. All authors wrote and edited the manuscript.

## Conflict of Interest

The authors declare that the research was conducted in the absence of any commercial or financial relationships that could be construed as a potential conflict of interest.
